# Phonetic accommodation in non-native directed speech supports L2 word learning and pronunciation

**DOI:** 10.1038/s41598-023-48648-7

**Published:** 2023-12-02

**Authors:** Giorgio Piazza, Marina Kalashnikova, Clara D. Martin

**Affiliations:** 1 Basque Center on Cognition, Brain and Language (BCBL), Mikeletegi Pasealekua, 69, 20009 Donostia-San Sebastián, Gipuzkoa Spain; 2 Ikerbasque, Basque Foundation for Science, Bilbao, Spain

**Keywords:** Psychology, Human behaviour

## Abstract

This study assessed whether Non-native Directed Speech (NNDS) facilitates second language (L2) learning, specifically L2 word learning and production. Spanish participants (N = 50) learned novel English words, presented either in NNDS or Native-Directed Speech (NDS), in two tasks: Recognition and Production. Recognition involved matching novel objects to their labels produced in NNDS or NDS. Production required participants to pronounce these objects’ labels. The novel words contained English vowel contrasts, which approximated Spanish vowel categories more (/i-ɪ/) or less (/ʌ-æ/). Participants in the NNDS group exhibited faster recognition of novel words, improved learning, and produced the /i-ɪ/ contrast with greater distinctiveness in comparison to the NDS group. Participants’ ability to discriminate the target vowel contrasts was also assessed before and after the tasks, with no improvement detected in the two groups. These findings support the didactic assumption of NNDS, indicating the relevance of the phonetic adaptations in this register for successful L2 acquisition.

## Introduction

Non-native Directed Speech (NNDS) is a clear speech register that native speakers use to address second language (L2) learners of their own language. NNDS is often studied in comparison with Native Directed Speech (NDS), which is the register used between native speakers without the intention of enhancing intelligibility^1^. NNDS has also been referred to as “L2 speech accommodation” because it is assumed to be the result of the speaker’s accommodation to the listener’s low L2 proficiency and learning needs (see^2–5^ for theoretical frameworks). In line with this, NNDS results in clear speech, and it is proposed to serve a didactic function by assisting learners to better understand, perceive, and pronounce their L2^6–8^. Piazza et al.^6^ proposed that the didactic function of NNDS comprises two related aspects: a *didactic purpose* and a *didactic impact*. The former is the function of producing clear speech to support L2 teaching, reflected in the acoustic features of NNDS, whereas the latter is the actual effect on L2 learning, perception, and production. While there is evidence for the didactic purpose indicating that speakers systematically adjust their speech production, resulting in clearer speech^8^, so far, the didactic impact of NNDS has never been directly explored. In the present study, we investigated whether L2 learners benefit from being exposed to NNDS by testing its didactic impact on perceiving, learning, and pronouncing L2 words.

### From high clarity to the didactic impact of NNDS

NNDS is characterised by speech adaptations to the non-native listener. Compared with NDS, such adaptations lead to the production of several acoustic features that enhance clarity of NNDS and potentially support L2 learning. The most typically studied features of NNDS are speech rate reduction and acoustic exaggeration of vowels, i.e., vowel hyperarticulation^6^. Vowel hyperarticulation is assumed to be the key acoustic feature that serves a didactic function in NNDS because it results in a clearer and more distinctive representation of vowel categories^9^. These features together are proposed to support speech perception, comprehension, and even production^1,6,10–12^. Indirect evidence that NNDS supports speech comprehension is provided by studies showing that listeners rate the intelligibility of NNDS higher than that of NDS. For instance,^8,13^ asked naïve native listeners to rate NNDS and NDS audio samples. NNDS was rated as clearer than NDS but less than other clear speech registers, like Lombard speech, which is a speech register produced to contrast background noise during native-native interactions^14–16^. Conversely, L2 learners have been reported to understand NNDS better than both NDS and Lombard speech, which is a register produced to contrast background noise during native-native interactions^17,18^. Lombard speech shares some acoustic features with NNDS, but manifested to different extents^6^. For instance, NNDS highlights phoneme differences to a greater extent than Lombard Speech^15,19^. In line with this,^20^ discovered that non-native listeners are not able to take advantage of Lombard Speech clarity as native speakers do, suggesting that Lombard Speech and NNDS fulfil different functions. Given that Lombard speech is not oriented to L2 learners, these seemingly conflicting results could be due to the lack of a didactic function (both purpose and impact) in Lombard speech^6^.

These rating findings indicate that the perception of NNDS and its enhancement of clarity differ between native and L2 learners, suggesting that there may be differences in how helpful NNDS may be for the two populations^21^. However, direct evidence showing that NNDS supports spoken word learning, recognition, or pronunciation in L2 learners is still missing. Few experiments have tested the efficacy of clear speech registers for word learning in adults. For instance^22,23^, found that Chinese Infant Directed Speech (IDS) helps non-native adult participants to learn words. IDS shares various acoustic features (including vowel hyperarticulation^8^) and proposed didactic function with NNDS, although these registers are intended for different addressees. Thus, one could expect that NNDS is particularly suited to support adults’ L2 learning. To test this assumption here, we investigated how L2 learners acquire perception and pronunciation of L2 words and phonemes when exposed to NNDS. In the following section we introduce the most relevant aspects of L2 word learning and the difficulties that novice L2 learners can face during this process.

### Aspects of auditory L2 word learning

#### Perception and assimilation

Initial L2 word learning is primarily mediated by the perception of novel phonemes^24–28^. L2 learners often have difficulties in discriminating phonetic contrasts that are not present in their L1 (both vowels and consonants). The relative difficulty in distinguishing L2 phonemes depends on the perceptual assimilation to the listener’s L1 phonology^26,29,30^. According to the Perceptual Assimilation Model for L2 (PAM-L2^29,31^), the most difficult situation for L2 perception is when the two L2 phonemes map onto a single native category (see also^26,32,33^ for alternative frameworks). In this case, the two L2 phonemes can either map equally to the native category (Single Category), or one phoneme can be a better fit than the other (Category Goodness). For Spanish learners of English, an example of Single Category is the vowel contrast /ʌ-æ/ (contained in words like *cup/cap*), comprised by two vowels that are not present in the Spanish phonemic inventory. In this case the pair of L2 vowels fall within the perceptual space of a single L1 vowel category (/a/), which makes it difficult to perceive the phonetic differences between the vowels^34–36^. Conversely, an example of Category Goodness for Spanish listeners is the vowel contrast /i-ɪ/ (contained in words like *sheep/ship*), in which the /i/ of *sheep* is a better instance of the Spanish /i/ than /ɪ/ (which is not present in the Spanish phonemic inventory). According to PAM-L2^29,31^, instances of Category Goodness are relatively easier to perceive than Single Category. To test this^34^, investigated late Spanish–English bilinguals ‘categorical perception of English vowel contrasts. Participants had particular difficulties recognizing /æ - ɑ/, /ʌ - ɑ/, and /ʌ-æ/ contrasts, whereas discrimination accuracy was higher for /ɪ - ɛ/ and /i-ɪ/ (see also^37^ for similar findings on perceptual ratings). Although accuracy was higher for /i-ɪ/, other studies found that Spanish late learners of English have difficulties discriminating this contrast^38,39^, and tend to perceive /i-ɪ/ vowels in a less categorical way than native listeners^35,40^. It is worth noting that this vowel contrast represents a special case of Category Goodness. That is, the English /i-ɪ/ contrast is not solely differentiated by spectral properties but also by duration cues, as the /i/ vowel is longer than the /ɪ/ vowel. Late Spanish–English bilinguals heavily rely on duration cues of this contrast to distinguish these two sounds, whereas native speakers of English and early bilinguals predominantly base their discrimination on spectral cues^35,38,39^. With English experience increasing, Spanish speakers tend to shift their reliance away from duration cues and to increasingly favour spectral cues in the discrimination of this contrast^38,41^.

There is broad consensus that experience (re)shapes L2 learners’ phoneme perception^24,41–43^. Flege et al.^41^ tested experienced and inexperienced L2 learners of various languages on synthetic /i-ɪ/ and /æ - ɛ/ continua and reported that the experienced group was more accurate than the inexperienced group at both perceiving and producing the vowel contrasts. This suggests that the perceptual system adapts to learning novel vowel contrasts and that perception can be changed with training^44–47^. However, it is not clear how much training is needed to observe such a change in the perception of phonological boundaries in L2 learners. Some studies found that perceptual change happens only in mid-to-high proficiency L2 learners^48^, whereas others found changes in low proficiency L2 learners within the duration of an experimental session^49,50^. Nevertheless, it is currently unknown whether such a perceptual change occurs in L2 learners after exposure to NNDS. L2 learners’ perceptual change of L2 phonemes is likely an important step in the learning process. Testing learning in the context of NNDS will also shed new light on the adaptation of phonological boundaries after short training in the L2.

In both types of phonetic assimilation discussed above, problems with the correct mapping of L2 phonemes hinders L2 learners from creating two distinct vowel categories. This determines the difficulty in perceiving and producing these vowels in a distinct manner^26,30^. So far, there is little evidence regarding the effectiveness of phonetic training for improving such mappings and phonological representations^51^, and, to our knowledge, there is no research on the effectiveness of NNDS in improving L2 perception. Therefore, in this study we focused on the learning process of both perception and production of L2 vowels and words in NNDS. We focused on the NNDS didactic impact for learning the two types of assimilation categories, Single Category and Category Goodness respectively, as realised by the /ʌ-æ/ and /i-ɪ/ English vowel contrasts. By doing this we aim to provide a well-rounded research approach for the study of the didactic impact of NNDS with a simulation of L2 learning of English words.

#### Production

The studies reviewed above all focused on L2 phoneme perception. However, this is just one, although fundamental, aspect of learning an L2, which also includes production^26^. L2 learners must deal with the challenge of correctly pronouncing novel words, and most adult L2 learners do not reach native-like pronunciation. Instead, speaking with a non-native accent, dependent on their L1, is common^52,53^. It is worth underlining that L2 learners’ most important objective is reaching comprehensible speech, rather than sounding like a native speaker (see^54,55^ for a discussion). Although L2 learners’ non-native pronunciation is expected, most naïve learners also have issues in distinguishing the pronunciation of L2 vowel contrasts^38,56^. This makes the two vowels difficult to distinguish, lowers intelligibility, and possibly leads to miscommunication. Thus, to accurately pronounce L2 vowels, phonetic differences between vowel categories must be learned. For this reason, we are also interested in investigating whether exposure to NNDS confers advantages in learning to pronounce words and vowels.

### The present study

L2 learners perceive NNDS to be clearer than NDS^17^, but to date, research assessing the impact of NNDS on L2 learning is not available^6^. To disclose the didactic impact of NNDS in the L2 learning process, there is need for research on the effect of exposure to NNDS on learning, perceiving, and producing L2 words and vowels. For this purpose, we recruited Spanish native listeners who were novice learners of English to participate in an online experiment. Participants were presented with novel objects and had to learn their associated English label. They were randomly assigned to a register group (NDS, NNDS) and asked to learn a set of 24 English pseudowords. All participants learned three types of novel words: (1) minimal pairs containing the /ʌ-æ/ contrast (like *guck*/*gack*), (2) minimal pairs containing the /i-ɪ/ contrast (like *deest/dist*), and (3) non-minimal pairs containing the /a/ and /u/ vowels (like *parg/phoon*), which were included as fillers to increase item variability. Participants were auditorily taught the associated label for each object in either NNDS or NDS. They were never presented with the spelling of the novel words. After this brief learning phase, participants completed three tasks to test word learning, word production, and vowel perception. Participants completed multiple blocks in each task, so that these tasks were part test and part training.

#### Recognition task

Participants were tested on the association between the (auditory) labels and novel objects. Accuracy and response times across blocks (*Block* factor) were compared between the NNDS and NDS groups.

#### Production task

Participants were presented with the previously learned objects, one by one, and were asked to pronounce their names. Response latencies across blocks (*Block* factor) were compared between the NNDS and NDS groups. We also computed phonetic accuracy—from the perspective of vowel distinctiveness—as means of the Euclidean distance (ED) within each vowel contrast (/i-ɪ/ and /ʌ-æ/) in participants’ productions.

#### Continuum discrimination task

Participants were administered two continuum categorical perception tests of the /i-ɪ/ and the /ʌ-æ/ contrasts embedded in familiar real words. We tested participants before the learning phase (pre-test) and after they completed both the Recognition and the Pronunciation tasks (post-test). That is, we investigated potential changes in participants’ ability to discriminate these vowels as a result of exposure to the sounds in NNDS or NDS registers.

Using these tasks, we were interested in answering the following questions:

1. Does NNDS enhance word learning as compared to NDS?
2. Does exposure to NNDS improve L2 vowel pronunciation distinction as compared to NDS?
3. Does exposure to NNDS as compared to NDS shape L2 vowel perception?

The Recognition and Production tasks aimed to answer the first question. In line with the assumption that NNDS yields a didactic impact on the process of L2 learning, we expected the NNDS group to learn the words and vowel sounds better than the NDS group. This would be revealed by a steeper learning curve across blocks and faster responses in the Recognition task. NNDS is also assumed to deliver articulatory information by providing L2 learners with exaggerated phonetic contrasts, which is not the case for NDS. Thus, in the Production task, we expected faster responses with a steeper learning curve in the NNDS group as compared to the NDS group. In addition, for Spanish participants, the /ʌ-æ/ contrast (Single Category, henceforth Single) is expected to be more difficult to produce than the /i-ɪ/ contrast (Category Goodness, henceforth Goodness)^24,29,41^. Thus, we expected participants to have lower accuracy and slower response times, in both tasks, for the Single than Goodness contrast.

The Production task also aimed to answer the second research question. As NNDS provides enhanced articulatory information, the NNDS group was expected to pronounce vowel contrasts (Single and Goodness) in a more distinct way than the NDS group, reflected by greater Euclidian Distance between vowels in the two contrasts. If this prediction was confirmed, it would imply that exposure to NNDS enhances the production of more intelligible vowel contrasts by increasing the distance (in formants) between vowels during pronunciation.

Lastly, the Continuum discrimination task aimed to answer the third research question. Previous research suggests that Spanish speakers struggle differentiating the vowel pairs used in this study. Native perception of vowels is quasi-categorical^57^, but non-native perception is not. Thus, both NDS and NNDS participants, with low levels of English knowledge, were not expected to show a clear perceptual boundary between the two target vowels in the pre-test. However, if NNDS enhances vowel discrimination, this may also transfer to previously known words. So, in the post-test, we expected only the NNDS group to show a more native-like perception of the two contrasts. This would suggest that NNDS induces adaptation in the listener’s L2 perceptual system after short training.

## Results

### Recognition task

**Figure 1:**
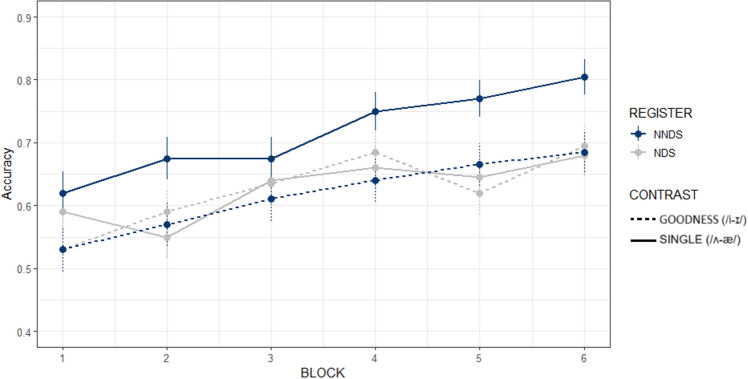
Recognition task. Accuracy across blocks, by *Register* (NNDS = Non-native Directed Speech, NDS = Native Directed Speech) and *Contrast* (SINGLE = novel words with the /ʌ-æ/ contrast; GOODNESS = novel words with the /i-ɪ/ contrast). Bars indicate SE.

This task aimed to investigate whether NNDS promotes L2 novel word learning. *Accuracy.* The final model indicated a significant effect of the *Block* factor’s linear term (β = 0.642, SE = 0.082, *p* < 0.001) but not quadratic term (β = − 0.0655, z = − 0.810, *p* = 0.419). Participants improved in accuracy linearly from 56.75% on average in Block 1 to 71.63% in Block 6. The main effects of *Register* (β = 0.274, z = 0.351, *p* = 0.436) and *Contrast* (β = − 0.258, SE = 0.139, *p* = 0.063) were not significant but their interaction was (β = 0.528, SE = 0.162, *p* < 0.001). The NNDS and NDS groups did not differ for the Single (β = − 0.010, SE = 0.357, *p* = 1) or the Goodness accuracy (β = − 0.538, SE = 0.358, *p* = 0.436). However, within contrasts, NNDS participants were more accurate in recognizing novel words containing the Goodness contrast than the Single contrast (β = 0.522, SE = 0.155, *p* = 0.004; see Fig. 1). Conversely in the NDS group this difference was not significant (β = − 0.006, SE = 0.152, *p* = 1). No other interactions were significant (see Data Availability).

#### Response latencies

**Figure 2:**
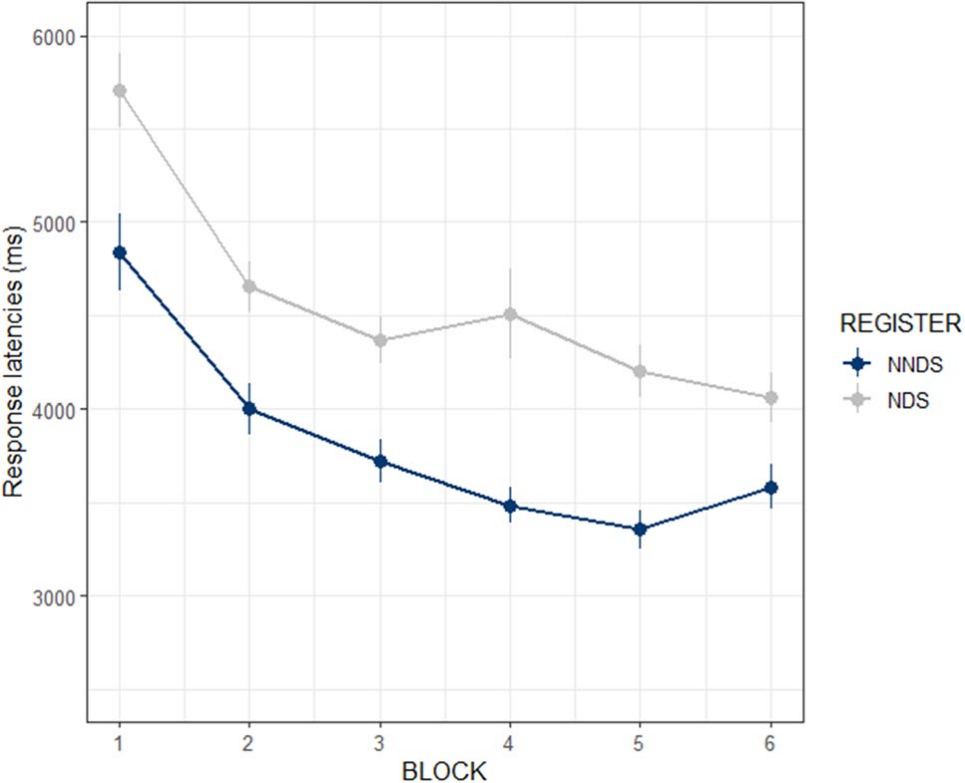
Recognition task. Response latencies across blocks by *Register* (NNDS = Non-native Directed Speech, NDS = Native Directed Speech) with responses collapsed across the two contrast types. Bars indicate SE.

The final model showed significant effects of linear (β = − 0.077, SE = 0.006, *p* < 0.001) and quadratic terms (β = 0.033, SE = 0.005, *p* < 0.001). This was due to a decrease in reaction time, from 6338 ms on average in the 1st block to 4834 ms in the 5th block, and then reached plateau performance in the 6th block (4892 ms on average). Also, the effect of *Register* was significant (β = 0.051, SE = 0.008, *p* < 0.001) with the NNDS group (3792 ms) responding overall faster than the NDS group (4551 ms; see Fig. 2). Conversely, the effect of *Contrast* (β = − 1.525e−04, SE = 0.008, *p* = 0.984) and any interaction did not reach significance.

### Production task

This task investigated whether NNDS promotes learning of novel words for production. One participant was excluded from the analyses due to very low production accuracy (~ 8%).

#### Response latencies

**Figure 3:**
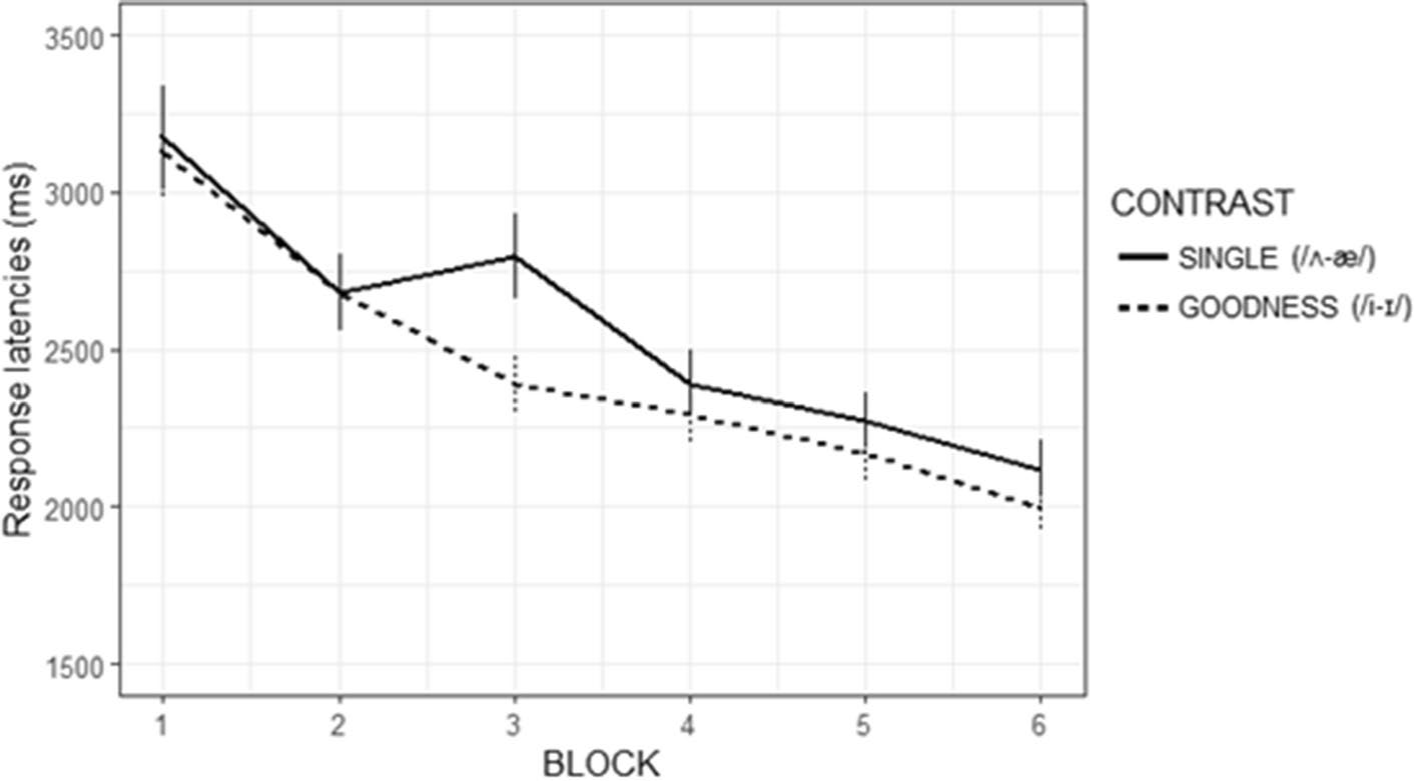
Production task. Response latencies across blocks by *Contrast* (SINGLE = novel words with the /ʌ-æ/ contrast; GOODNESS = novel words with the /i-ɪ/ contrast). Bars indicate SE. These data are collapsed across registers.

The final model yielded a significant quadratic term (β = 0.046, SE = 0.018, *p* = 0.004) but not linear term (β = 0.014, SE = 0.017, *p* = 0.414), indicating that participants’ response latencies across blocks best fitted a parabola shape. The *Contrast* factor showed a significant effect (β = − 0.049, SE = 0.024, *p* = 0.037) reflecting overall shorter latencies in producing the Goodness contrast (1946 ms) than the Single contrast (2408 ms; see Fig. 3), particularly form Block 3 onwards. Conversely, the *Register* factor (β = − 0.022, SE = 0.069, *p* = 0.757) was not significant and neither were any interactions.

#### Euclidean distance

**Figure 4:**
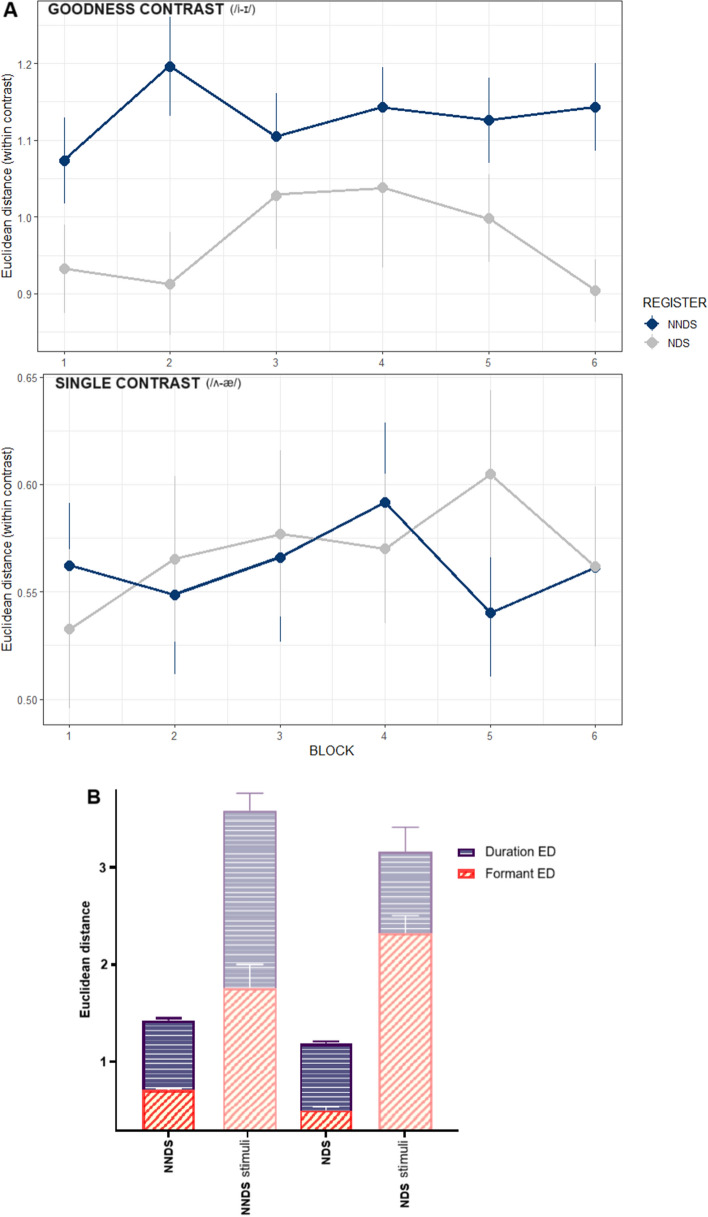
Production task. (**A**) Normalized Euclidean distance (ED) by *Register* (NNDS = Non-native Directed Speech, NDS = Native Directed Speech) and *Contrast* (SINGLE = novel words with the /ʌ-æ/ contrast; GOODNESS = novel words with the /i-ɪ/ contrast). Bars indicate SE. (**B**) Participants’ composite ED (formants and duration) and stimuli ED of the Goodness contrast by *Register* NNDS = Non-native Directed Speech, NDS = Native Directed Speech). Bars indicate SE.

The analysis of EDs assessed whether the exposure to NNDS improved category distinction as compared to NDS. For this purpose, we computed ED of the two contrasts, Goodness (/i-ɪ/) and Single (/ʌ-æ/). The EDs were computed differently for each contrast: accounting for formant and duration distance for Goodness (/i-ɪ/), and formants only for Single (/ʌ-æ/) (see Method for more details). These were separately investigated in two models. Each model included the *Block* and *Register* factors. The final model for the Single contrast did not show any significant effect or interactions (Register: β = 0.011, SE = 0.043, *p* = 0.806; linear term*:* β = 0.004, SE = 0.031, *p* = 0.903*;* quadratic term*:* β = − 0.034, SE = 0.030, *p* = 0.267). The final model for the Goodness contrast indicated a main effect of *Register* (β = − 0.183, SE = 0.061, *p* = 0.005) but no effect of linear (β = − 0.080, SE = 0.056, *p* = 0.152) or quadratic terms (β = 0.005, SE = 0.057, *p* = 0.934) or interactions (see Data Availability). The NNDS group produced the vowels in this contrast more distinctly than the NDS group (Euclidean Distance NNDS = 0.987; NDS = 0.913), without substantial changes across the 6 blocks (see Fig. 4). Given the significant effect in the Goodness contrast, Fig. 4B provides a comprehensive view of the participants' production in this contrast. This figure shows both the composite ED of participants' production (including formants and duration ED) in both the NNDS and NDS groups and the reference ED values of the stimuli they were exposed to (Goodness contrast only).

#### Continuum discrimination task

**Figure 5:**
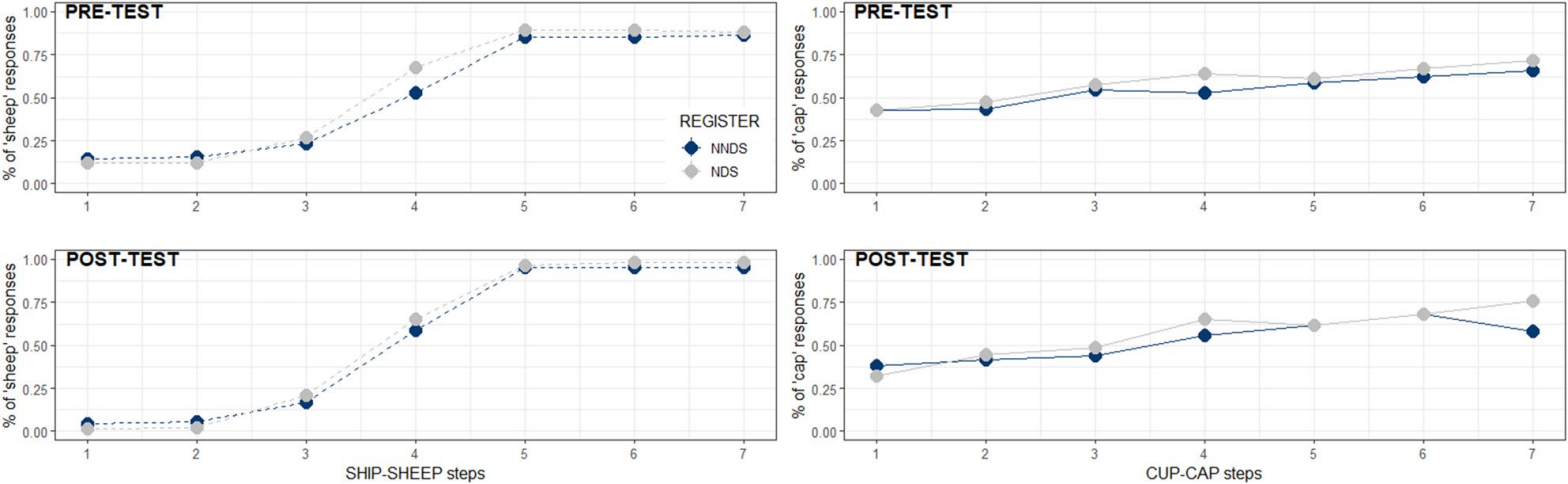
Continuum discrimination. Average percentage of ‘sheep’ choices (on the left) and of ‘cap’ choices (on the right) across the seven-step continuum by *Register* (NNDS = Non-native Directed Speech, NDS = Native Directed Speech) and by *Exposure* (pre-test and post-test). Bars indicate SE.

This task assessed whether the brief exposure to the target vowel sounds in NNDS or NDS induced changes in the participants’ L2 perceptual system that transferred to real English words. No significant main effects or interactions were found in the final model for either the sheep-ship continuum (*Register*, β = 0.181, SE = 0.200, *p* = 0.365, *Exposure,* β = 0.046, SE = 0.088, *p* = 0.599) or the cup-cap continuum (Register, β = 0.264, SE = 0.280, *p* = 0.346; Exposure, β = − 0.085, SE = 0.092, *p* = 0.355, see Fig. 5).

## Discussion

Previous literature has assumed that NNDS is endowed with a didactic purpose—reflected in the acoustic features of NNDS—and a didactic impact^6–8,58^. Such a didactic impact would support L2 learners both in comprehension and production. However, so far, whether L2 learners’ perceptual and production learning is promoted by exposure to NNDS remained unknown. We addressed these questions by conducting an online experiment where two groups of L2 learners of English (Spanish L1) learned the association between novel objects and novel English words pronounced in either NNDS or NDS. Perception and learning of English vowel contrasts (/i-ɪ/ = Goodness, /ʌ-æ/ = Single), which are absent in the Spanish phonological inventory, was assessed. In order to investigate whether NNDS yields learning benefits in the production of novel words and vowels, participants’ latency and vowel production were also measured. We predicted that the group exposed to NNDS would learn to perceive novel words and pronounce vowel contrasts more successfully and faster than the NDS group.

### NNDS benefits

The present study provides the first evidence for the benefits of NNDS on L2 learning. That is, NNDS participants were better at perceiving L2 novel words as compared to NDS participants. Such a benefit was mainly shown in the Recognition task results, which indicated that the NNDS group responded faster than the NDS group in recognising novel words (both vowel contrasts). This represents evidence in support of the didactic function hypothesis of NNDS and speech accommodation theories^2–6,8,17^.

### NNDS benefit depends on properties of the speech contrasts to be learned

Our results also provide evidence that NNDS effects are qualified by the properties of the speech contrasts to be learned, and how they relate to listeners’ L1^29^. That is, NNDS benefits were particularly pronounced for the Goodness contrast (/i-ɪ/). For example, Recognition accuracy of the NNDS group was higher for the Goodness than the Single contrast, whereas there was no such improvement in the NDS group. This suggests that even though the NNDS group did not show overall better accuracy than the NDS group for both contrasts, their exposure to NNDS promoted recognition of words including the Goodness contrast. On the other hand, Production results showed that NNDS delivered articulatory information that improved L2 pronunciation distinctiveness, but for the Goodness contrast only (larger /i-ɪ/ distance in their production; in line with^29^). This result suggests that NNDS provides articulatory information to the listeners, who use such cues to pronounce distinct vowels and, thus, promote intelligibility of their productions.

These findings are probably due to the acoustic features of NNDS, which enhance the differences between vowels. The NNDS novel words containing the Goodness contrast were produced (by a native speaker who recorded the stimuli) with greater /i-ɪ/ duration differences and reduced formant ED than the same novel words pronounced in NDS (see Material and Appendix 2 in the Supplementary Material). Participants’ performance was in line with previous literature that reported Spanish listeners to be particularly sensitive to duration differences between L2 vowels^24,35,38,39,59^. This also indicates that NNDS duration cues (directed to Spanish listeners) are particularly suited to enhance L2 learners’ discrimination of the /i-ɪ/ vowel contrast rather than contrasts signalled by formant value information. Research has suggested that such cues are intuitively produced by native speakers to support communication with L2 learners^2,6,8,58^; see also Appendix 2 in the Supplementary Material). Here, we show that these duration cues also support word learning, bearing a didactic impact for L2 learners. Our results do not enable us to exclude a beneficial role of NNDS in supporting learning of Single contrast vowels, but suggest that such effect may be weaker and require more exposure to lead to detectable improvements. Lastly, production latency results showed that participants were faster to respond to Goodness than Single contrast words, regardless of the Register group. This finding does not relate to our focus on differences between NNDS and NDS, but it is still interesting because it confirms that the Goodness contrast used here is easier to discriminate than the Single contrast for our participants, as we discuss below.

### Theories of second language acquisition that explain the NNDS benefit

The asymmetrical benefit we observed between Goodness and Single contrasts is in line with PAM-L2, which claims that Goodness contrast phonemes are more easily recognized and pronounced than Single contrast phonemes^29,31^. Other second language acquisition accounts also provide explanations for this learning asymmetry, such as the Native Language Magnet Theory^60,61^, the Speech Learning Model^26,32^, the Contrastive Analysis Hypothesis^33,62,63^, and the Input Hypothesis (part of the Monitor Model;^64–66^. For instance, the Input Hypothesis assumes that L2 learners acquire language when they are exposed to comprehensible input. This refers to language input containing previously acquired elements and new instances that are slightly beyond L2 learners’ current level of proficiency. Accordingly, when participants learned the Goodness contrast, /i/ represented the known element and /ɪ/ the new instance to be acquired. Conversely, the Single contrast was far beyond participants’ proficiency level for substantial improvement. The disparity in learning was further reinforced by the types of cues provided. In the case of the Goodness contrast, one cue was duration, a feature that is familiar to Spanish learners of English, which made the input more comprehensible. Conversely, the formants of the Single contrast proved to be challenging to perceive and learn, contributing to the difficulty in acquiring it. Thus, participants in both NNDS and NDS groups may have received comprehensible input that facilitated overall learning of the Goodness contrast, though this learning was more successful in NNDS.

It was also the case that participants showed greater improvement in learning the Goodness contrast than the Single contrast if exposed to NNDS rather than NDS. But the above-described accounts, including the Input Hypothesis, do not consider such an interaction between Register and Contrast. For instance, the Input Hypothesis does not specifically address the learning of phonetic contrasts or pronunciation and does not fully explain the observed benefit in NNDS compared to NDS. The significant improvement in learning observed in NNDS compared to NDS suggests that there may be additional factors at play.

To provide a more comprehensive explanation, it may be necessary to incorporate additional theories and factors. The interaction between Register and Contrast can be further explained by adding a complementary socio-cognitive factor^67^ to the previous models, which would provide a combined framework that can explain this advantage for the Goodness contrast in NNDS ^6,8,9^. The socio-cognitive theory of second language acquisition claims that L2 learning is a natural and adaptive process of ecological alignment^67–69^. In fact, our results reveal that learners adapt their perception and production of L2 novel words to the social environment (i.e., learning differs depending on speech adaptation of the speaker/teacher). This suggests that NNDS is a socially mediated promoter of phoneme category distinction and acquisition.

### NNDS benefit depends on the modality and task demands

NNDS seems to be a suitable tool for teaching a second language, which supports L2 learners’ performance, both in recognition and production. However, the present study also revealed that this overall L2 support differs depending on the modality (i.e., word recognition vs. production). We observed better recognition and production performance in the NNDS than NDS group (as for the Goodness contrast), but the production benefit was limited to greater distinctiveness in vowel contrast pronunciation (ED measure). In sum, the NNDS benefit was visible in faster word recognition (and higher production intelligibility) but not in faster word production. It could be that NNDS is beneficial for word production speed as well, but that longer training would be needed to observe those effects on production^70^. This assumption is in line with previous literature reporting that, when learning linguistic elements, comprehension precedes production learning^71–73^.

However, it is worth noting that the Production task was always carried out after the Recognition task. Participants first learned to perceive the differences between vowels and novel words, and only afterwards were asked to produce them. We argue that this could be the main cause of the observed disadvantage in the production of /ɪ/ of the NDS participants. During the Recognition task, NDS participants were exposed to novel words containing the /i-ɪ/ contrast in which the duration cue (/ɪ/ shorter than /i/) was reduced as compared to the NNDS group. We think this absence of clear duration cues might have impaired accurate perception (and thus learning) of the Goodness contrast. Thus, NDS participants carried over this disadvantage to the Production task, where they could not improve their production^26^. Nonetheless, NNDS participants, who were exposed to reduced /i-ɪ/ formant ED as compared to NDS, instead produced wider /i-ɪ/ formant ED than NDS participants (see Fig. 4B and Appendix 2 in Supplementary Material). By presenting participants and target vowels composite EDs data side by side, Fig. 4B enabled us to assess the differential impact of formant ED and duration ED of the stimuli on participants’ production ED. This leads to two important observations: (a) for Spanish listeners, duration cues are particularly relevant for learning the /i-ɪ/ contrast, and this affects vowel formant production learning as well; (b) the NNDS production benefit does not simply derive from mimicking perceived target phonemes and from being exposed to wider vocalic ED. In fact, NNDS participants produced more distinct vowels without mimicking phonemes they were exposed to. This reveals that exposure to NNDS enhances L2 speakers’ distinctiveness production beyond mimicking—a strong argument in favour of the didactic purpose of NNDS.

An important consideration is that the present study used an online method to collect participants’ responses. Several studies have addressed the question of whether online experiments provide reliable results and revealed that chronometric experiments for speech production can be implemented online without information loss^74–78^. Thus, we are confident in sustaining that the differences we found between speech register groups were genuine and not driven by the online setting. However, future research should run similar experiments in a laboratory to dispel any doubts that the benefit derived from the exposure to NNDS differs online and onsite.

### NNDS does not induce changes in L2 sound phonetic boundaries (after short training)

Lastly, we found that the effects of NNDS exposure did not—at least in this study—change participants’ phonetic boundaries of the /i-ɪ/ and /ʌ-æ/ contrasts: phonetic boundaries did not become more native-like despite the improvement in both word recognition and production. In the Continuum discrimination task, we expected to find an adaptation of the phonetic boundaries for both continua (*sheep-ship* and *cup-cap*) in the NNDS group’s post-test. However, we did not find any difference between the two groups, nor between pre-test and post-test in both vowel continua. This means that the two groups did not significantly differ for initial perception of the two vowel contrasts, and that neither of the two changed their phonetic boundaries in the post-test. We expected to observe this pattern in the NDS, but not the NNDS group who were exposed to more distinct tokens of the categories forming the two phonemic contrasts. According to studies on distributional learning, adult listeners should be more successful in acquiring categories in this case compared to NDS, where the category tokens occur close together, making it more difficult to differentiate category distributions^70,79–81^. Previous research suggests that adaptation of phonetic boundaries can happen within a single experimental session^49,50^, whereas other research points that longer exposure and experience is needed^48^. Our result aligns with the latter proposal. However, research reported that phonetic adaptation within a single experimental session is visible at the neurophysiological level^82^. We cannot exclude, therefore, that NNDS induces phonetic adaptation after short training, but it is not detectable at the behavioural level, with the particular task and stimuli we used. Thus, further research (both using behavioural and neurophysiological methods) is needed to address this point.

To summarise, this study provides new insights on the process of learning an L2 after exposure to NNDS and makes a step forward to understanding the precise mechanisms involved in L2 teaching and learning. We found that NNDS has an impact on learning L2 words for recognition and production, but (especially) improvements in production intelligibility (vowel distinctiveness) depend on the relationships between the phonemes to be learned and learner’s L1 phonemic categories. It is important to underline that, in this study, participants were exposed to NNDS (or NDS) for a very short period (< 2 h); hence, it is probable that more benefits would derive from extended exposure to NNDS (e.g., classroom teaching). These findings and future research on more prolonged exposure to NNDS are fundamental to building models of L2 communication and learning. This research is particularly relevant given that communication between native and non-native speakers is becoming ever more frequent in our increasingly multicultural and multilingual societies.

## Method

### Participants

We recruited 50 native Spanish participants with a low-to-mid level of English knowledge, aged 18–40. Participants were recruited following an individual interview with an expert linguist, who assessed their English level and assigned marks from 1.0 to 5.0 (1.0 = low; 5.0 = native-like). In the interview, fluency, vocabulary, grammar, and pronunciation were evaluated, and then combined into an overall mark. We only recruited participants who obtained an overall mark between 1.0 and 3.0 (NDS group: M_mark_ = 1.8, SD = 0.45, NNDS group: M_mark_ = 1.9, SD = 0.32). The participants were randomly assigned to one of two groups (25 participants each), exposed to either NDS or NNDS (NDS group: M_age_ = 26.76 years, SD = 6.55, Male = 3; NNDS group: M_age_ = 27.36 years, SD = 6.48, Male = 3). In addition, at the end of the experimental session, participants were asked to carry out a Raven matrices test and a pseudoword repetition task in Spanish, used as indices of participants’ non-verbal IQ and phonological memory^83,84^ (see Appendix 4 for a description of these tasks). All participants signed an informed consent form before starting the experimental procedure, and the study was approved by the Basque Center on Cognition, Brain and Language (BCBL) Ethics Committee and conducted in accordance with the relevant guidelines and regulations. Participants were paid 20 euros for taking part in the study.

Bayesian analyses showed that the two groups did not significantly differ in age, English proficiency, non-verbal IQ, and phonological memory. Two-tailed analyses with Cauchy prior distribution (scale of γ = 0.707) revealed that age, proficiency, IQ, and phonological memory of the two groups were respectively (Bayes factors, BF_01_) 3.39, 2.25, 3.05, and 3.53 times more likely under the null than the alternative hypothesis.

### Material

Empirical evidence on the realisation of vowels other than /a/,/i/,/u/ (e.g., /ɪ/, /ʌ/, /æ/) in NNDS is limited in the literature. For this reason, we first ran a pilot study to assess matrices of NNDS adaptation on /ɪ/, /ʌ/, /æ/ vowels. We recruited five native speakers of English (British accent), who were (or had been) teachers of English with Spanish speaking students. We report the results and description of this preliminary study in Appendix 1. Below, the materials used in the three tasks are described.

#### Recognition task and production task

For the present study, we created 16 novel words containing the /i-ɪ/ (e.g., [di:st - dɪst]) and /ʌ-æ/ contrasts (e.g., [gʌk – gæk]). The novel words for both vowel contrasts were minimal pairs, so that participants had to rely on the target vowels to distinguish the words. To increase item variability, we also created 8 novel words containing the /a/ and /u/ vowels (not forming minimal pairs) that served as fillers (e.g., [p^h^a:g – fu:n]; see Appendix 2 for the full list of experimental stimuli). The 24 novel words (16 targets + 8 fillers) were either monosyllabic or disyllabic to increase variability (that simulates naturalistic learning) and to reduce task difficulty (that would have emerged from using only monosyllabic and thus highly similar items). A set of 24 novel objects was selected to match the 16 target novel words and 8 filler words. The images were taken from the^85^ novel object database and represented unknown objects and unfamiliar tools. To create the object-word pairings while avoiding any effects derived from specific relations between words and objects in our stimuli, we created 3 lists of pseudo-random associations, and the presentation of these word-object lists was counterbalanced across participants.

The stimuli were recorded by a female native speaker of British English. This speaker was chosen from the 5 speakers who participated in the pilot study as best representing the observed preliminary results (see Appendix 2; wider vocalic area, longer sentence duration, larger /ʌ-æ/ ED and /i-ɪ/ duration difference). This speaker produced novel words in NNDS with wider vocalic area (+ 187%), longer sentence duration (M_NNDS_ = 3640 ms, M_NDS_ = 3561 ms), greater /ʌ-æ/ ED (M_NNDS_ = 358.10 Hz^2^, M_NDS_ = 161.96 Hz^2^, and larger /i-ɪ/ duration difference (M_NNDS_ = 15 ms, M_NDS_ = 4 ms) than in NDS. Conversely, she produced smaller /i-ɪ/ ED in NNDS than NDS (M_NNDS_ = 933.88 Hz^2^, M_NDS_ = 1169.25 Hz^2^). All stimuli were normalised for intensity and used in both the Recognition and the Production task.

#### Continuum discrimination task

A female native speaker of British English, who did not record the stimuli for the other tasks, was recorded while producing the words *sheep*, *ship*, *cup*, *cap*. These recordings were used to create two seven-step continua. The sheep-ship continuum was created by gradually changing the formants and the length of the target vowels. The cup-cap continuum was created by solely changing the formants of the target vowels as this contrast is not marked by vowel duration^24,35^. Based on the continua, we created 7 isolated instances of words from sheep to ship and from cup to cap that were used in this task.

### Procedure

The experiment was administered online via PennController for Ibex^86^, which is a JavaScript-based platform. During the session, participants remained connected with the experimenter via Zoom™, but video streaming was always disabled. This allowed the experimenter to verify that participants’ microphone worked properly and that they stayed focused on the task, without the participants feeling observed during the session. We asked participants to wear headphones and a head-mounted microphone if available, but any type of microphone with acceptable quality was allowed. Before the start of the experiment, participants recorded and played back their own voice to self-check audio quality. Participants’ compliance was confirmed using a screening test^87^. After that, the experimental session followed this order: Continuum discrimination task (pre-test), Familiarisation phase, Recognition task, Production task, Continuum discrimination task (post-test), Raven matrices test, Pseudoword repetition task. Each session lasted about 95–100 min.

#### Continuum discrimination

The task began by displaying two images on the screen, one at a time (either a sheep and a ship or a cup and a cap, in counterbalanced order across participants). For each image, participants were presented with an auditory recording of the image’s name pronounced in NDS. Then, the task started, and participants used the mouse to click a button on the centre of the screen to listen to the stimuli. They were presented with one sound of the continuum at a time (in a random order). The two pictures previously displayed (a sheep and a ship or a cup and a cap) were presented on the screen and participants were asked to click on the picture corresponding to the word they heard. Each endpoint and mid-step word (7 in total) were repeated 6 times (42 trials per contrast). After completing the block corresponding to the first two images (e.g., sheep and ship), the same procedure was followed for the other minimal pair (e.g., cup and cap). Both pre-test and post-test followed the exact same procedure.

#### Familiarisation phase

The object-word pairs were presented once during this phase. Participants were exposed to the novel objects presented together with the auditory version of their name, embedded in a carrier phrase (e.g., “this is a *deest*”). The images of the objects were presented one at the time and after 250 ms the phrase containing the label was played. Next, a button appeared on the screen and the participants clicked on it to proceed to the next object. Each sentence was pronounced in either NNDS or NDS, depending on the participants’ group allocation. Target and filler novel words were presented in a random order and no response was required by participants (passive learning task). It is worth noting that the same novel words were used in both groups (but presented in either NNDS or NDS), so that differences across novel words should not strongly influence the results.

#### Recognition task

Participants saw images of 4 objects on the screen and heard a sentence used in the familiarisation phase (e.g., “this is a *deest*”). The 4 objects comprised the target object (e.g., the referent of deest), a competitor (e.g., which served as a referent of dist on another trial) and two distractors (e.g., which served as referents of *gack* and *phoon* on other trials*)*. Participants used the mouse to click a button on the centre of the screen to hear the cue-sentence. Then, the objects were displayed on the screen until participants provided a response by clicking on one of the 4 objects. As soon as they did so, all the objects disappeared and the correct one was displayed on the centre of the screen for 2500 ms. This provided feedback on the correct answer to participants. Each block included 24 trials (16 experimental trials + 8 fillers) and participants were exposed to 6 blocks in a row (total number: 96 target trials + 48 fillers). In this way, each block served both as a test and further training of the novel words. Stimuli presentation was pseudorandomized to prevent the same target vowel appearing more than twice in a row.

#### Production task

Participants were presented with the same 24 objects from the recognition task. The objects were displayed one at a time on the screen and the participants were asked to name each of them. As soon as an object was displayed on the screen, the browser started recording participants’ oral responses. The object remained on the screen until participants clicked the button ‘Send your response’. The microphone continued recording for 500 ms after the response was sent to avoid any responses being trimmed by an early button press. After sending their response, participants heard the novel word embedded in the carrier phrase, as in the recognition task, which served as feedback. Then, the next trial began by displaying a new object on the screen. This procedure was repeated until all the object-word pairs were presented (in random order) and repeated in 6 consecutive blocks (96 target trials + 48 fillers).

### Measures and statistical analysis

#### Recognition task

For this task we extracted (1) response accuracy across the 6 blocks. Offline, scores of 0 and 1 were assigned respectively to incorrect and correct responses. We also measured (2) response latencies across blocks. Latencies were measured from the moment the cue-sentence finished playing to the moment participants provided an answer. Only correct answers were included in the latency analysis.

#### Production task

We measured (1) response latencies across blocks, measured from the object presentation until participants orally responded. Furthermore, based on the values of the first (F1) and second (F2) formants and vowel duration, we computed the Euclidean distance within participants’ Goodness contrast productions (/i-ɪ/), as the three features together differentiate the two vowels of the contrast. On the other hand, vowels of the Single contrast (ʌ–æ) are differentiated by formants only; that is, there is no reason to expect that participants employ duration to distinguish the two vowels. Thus, we computed the Euclidean distance within participants’ Single contrast productions by including F1 and F2 measures. Thus, for the Single contrast we computed the ED based on F1 and F2 only. In addition, participants’ vowel productions (were normalised using the Lobanov method^88^. This method uses a log-mean method to normalise the formant values and computes a single grand mean for all participants, based on their vocalic triangle. Such an approach was used to prevent participants’ physiological differences from driving the observed effects.

All incorrect responses or that—despite some similarity with the target—clearly pointed at a distractor were excluded from the analyses. For example, if a participant said [pi:fəl] for the object associated with the novel word [pi:v], their response was considered incorrect and excluded from analyses of latency and the two EDs because it pointed at the distractor [bi:fəl]. The excluded trials represented 39.58% of the total responses. A total of 2900 trials were kept for analyses: 1559 in NNDS and 1341 in NDS (BF_01_ = 1.89, anecdotal evidence for H_0_).

The dependent variables of the Recognition and Production tasks were independently analysed using growth curve analysis (GCA) models^89,90^ fitted in R (*lme4* package;^91^; see Appendix 3 for a list of the models). This technique is explicitly designed to assess changes over time at group and individual levels. GCA allowed us to add to the models the linear and quadratic polynomial terms to account for the overall slope change and the curvature of the observed effects. The linear term reflects the overall slope, and the quadratic term reflects the curvature (i.e., change in slope across learning blocks). Thus, the 6 blocks were added to the model as *Block* factor, including linear and/or quadratic terms depending on the best model fit. The *Register* (NNDS and NDS) and *Contrast* (Single and Goodness contrasts) factors, together with the *Block* factor, were added to the models as fixed effects (unless otherwise specified). Subject and novel words were included as random effects. Other predictors were considered only if they improved the model fit (see Appendix 3 for a list of the final models). Starting with the minimal structure, various models were created; the final models were chosen according to the best fit indicated by the *Performance* package in R^92^. For all models, we set a priori sum contrasts so that within *Register,* − 0.5 was assigned to NDS and + 0.5 to NNDS, whereas within the *Contrast* factor, − 0.5 was assigned to Category Goodness and + 0.5 to Single Category^93^. Response latencies were transformed using the Box-Cox method^94^. Conversely, accuracy of the Recognition task was tested by fitting GCA with generalised linear mixed-effects (*glmer*) models (binomial family). Both measures of ED were tested in two separate models (one for each contrast: Single and Goodness).

#### Continuum discrimination

For this task, we used a generalised linear mixed effect model (binomial family) to compare vowel discrimination between the pre-test and the post-test (*Exposure* factor) and between the two speech register groups. We did not include polynomial terms because GCA did not apply for this variable. Ship/sheep and cup/cap continua were tested in separate models.

For all tasks, model significance was tested with the *lmerTest* Package^95^ and interactions between main effects were explored by running post-hoc analyses in the *emmeans* package^96^ with Tukey HSD correction for multiple comparisons. Given the number of interactions tested in each model, below we report only significant interactions; all results, including non-significant results are reported in the Data Availability.

### Supplementary Information

Supplementary Information.

## Data Availability

Material, data, experiment script, analysis code, and non-significant results can be found at https://osf.io/xtky5/?view_only=4ec02c26bd084296b088780811ebbb07. A list of the material, supplementary images, and statistical formula can be found in Appendix 1, 2 and 3.
