# Supplementary material for: Phonetic accommodation in non-native directed speech supports L2 word learning and pronunciation
Source: Sci Rep. 2023 Dec 2;13:21282. doi: 10.1038/s41598-023-48648-7 (PMC10693623; doi:10.1038/s41598-023-48648-7)
Supplement: Supplementary file 1 — Supplementary Information. [file 41598_2023_48648_MOESM1_ESM.pdf]

Appendix 1

NNDS of English vowel contrasts: five English speakers.

Previous studies on NNDS suggest that this register is the result of speech adaptation to non-native listeners' learning needs. Yet, empirical evidence is limited to hyperarticulation of the vowel triangle that captures production of the three corner vowels /a/, /i/, and /u/. This limits generalisation of NNDS impact on learning, especially for a language with rich vowel inventory like English [1]. Thus, prior to the actual experiment, we aimed to assess the Euclidean distance between vowels of the /i-ɪ/ and /ʌ-æ/ contrasts and the vowel duration difference for the /i-ɪ/ contrast as index of NNDS speech adaptation. For this purpose, we recruited five native speakers of English (British accent) (M_{age} = 32.3 y.o., Male = 4), who were (or had been) teachers of English for Spanish speaking students. The speakers carried out an online experiment, where they were asked to speak as if they were addressing a native listener of English or a Spanish native L2 learner of English. These instructions were aimed to elicit two registers, NDS and NNDS, respectively. Before the start of each register-block, speakers listened to a short introduction about the listener. Then, they were instructed to pretend they were speaking to that person. The speakers read English words appearing on the screen and were asked to say "this is a [word on the screen]". After each production, they pressed 'Enter' to proceed to the next trial.

We selected 48 monosyllabic and disyllabic real English words containing the 4 vowels of interest (/i/, /ɪ/, /ʌ, /æ/), which comprised minimal pairs differing by only the target vowels. There were 8 word-minimal pairs containing the /i-ɪ/ contrast (total 16 words like *sheep-ship*) and 8 word-minimal pairs containing the /ʌ-æ/ contrast (total 16 words like *cup-cap*). To these, we added 16 words containing the /a/ and /u/ vowels (8 each), which did not form minimal pairs. Those words were considered as fillers and were added in order to avoid speakers being conscious that the two vowel contrasts were of special interest for us. Words were presented in pseudorandomized order, in all caps and in the centre of the screen. Speakers repeated each word 6 times (3 addressing each listener) for a total number of 288 trials.

From the resulting production data, we first examined the vocalic space of the /a/, /i/, and /u/ triangle of the two registers. Also, we measured the duration of sentences (“this is a…”) speakers produced. These two measures allowed us to assess whether speakers produced NNDS vocalic triangle and speech rate in line with previous literature (e.g., [1], [2] [3]). Next, we focused on the minimal pairs to measure the Euclidean distance of the /i-ɪ/ and /ʌ-æ/ contrasts and the duration difference of the /i-ɪ/ contrast (duration of /i/ - duration of /ɪ/). Euclidean distance was calculated by projecting on a cartesian plane the first (F1) and second (F2) vowel formants of the speakers’ production. If speakers adapt to the listeners needs and produce NNDS to support L2 learning, we expected the speakers to produce 1) a wider vocalic triangle 2) lower speech rate 2) greater /i/ minus /ɪ/ duration difference 3) greater Euclidean distance between /ʌ-æ/ and between /i-ɪ/ in NNDS as compared to NDS.

In line with the literature, non-parametric Wilcoxon tests revealed that NNDS was produced with a wider vocalic triangle (+30.6%)[1] and lower speech rate compared to NDS (W = 856426, p < .0001). Also, non-parametric Wilcoxon tests revealed that speakers produced greater /ʌ-æ/ Euclidean distance (W = 7057, p = .016), and /i-ɪ/ duration difference (W = 8213, p = .001) in NNDS than NDS. Unexpectedly, the /i-ɪ/ Euclidean distance was greater in NDS than NNDS (W = 4235, p = .038). These results suggest that speakers emphasise duration difference between /i-ɪ/, and Euclidean distance between /ʌ-æ/, when producing NNDS. These results were used as the bases for the novel word stimuli of the present study. For recording the stimuli, we selected the speaker who produced NNDS with more hyperarticulated vowels, greater duration difference between /i-ɪ/, greater /ʌ-æ/ Euclidean distance and with smaller /i-ɪ/ Euclidean distance than NDS.

Appendix 2

List of experimental stimuli

BEEFUL	BIFUL	CABBON	CUBBON
DEEST	DIST	DAGMET	DUGMET
GHEEDEN	GHIDEN	GACK	GUCK

[1] Statistical analysis for the vocalic space could not be run due to the low number of observations: we extracted only one vocalic space value per each subject and condition.

PEEV	PIV	TASS	TUSS

List of fillers

POOTON	TARPEL
SOOTIC	FARSIK
TOOD	SARN
PHOON	PARG

Appendix 3

(GCA) Mixed-effect model formulas

1. *Recognition accuracy*

model <- glmer(ACCURACY ~ (ot1+ot2)*REGISTER*CONTRAST+(ot1|IQ:SUBJ)+(1|WORD),family = "binomial", data = data*)*

2. *Recognition RT*

model <- lmer(RT_bc ~ (ot1+ot2)*REGISTER*CONTRAST+(ot1+ot2|WORD:SUBJ), data =data,REML = F)

3. *Production RT*

model <- lmer(RT_bc ~ (ot1+ot2)*REGISTER*CONTRAST+(1|SUBJ)+(1|PHON:WORD), data = data, REML = F)

4. *Euclidean distance*

model_Goodness_c <- lmer(EUCLIDEAN ~ (ot1+ot2)*REGISTER+(1|ot1:SUBJ)+(1|PHON:WORD), data = Goodness, REML = F)

model_Single_c <- lmer(EUCLIDEAN ~ (ot1+ot2)*REGISTER+(1|SUBJ)+(1|PHON:WORD), data = Single, REML = F)

5. *Logistic regression (sheep-ship/cup-cap)*

model <- glmer(CHOICE~ EXPOSURE*REGISTER+(1|SUBJ)+(1|CONT), data = data, family = "binomial")

IQ = Participants' non-verbal IQ scores

PHON = Phonological memory scores

WORD = novel word/item.

Appendix 4

Raven matrices test and pseudoword repetition: materials and procedure.

Participants' non-verbal IQ was assessed using the Raven matrices from the Kaufman Brief Intelligence Test (KBIT; [4]). Participants completed as many sequences as they could

in 6 minutes. Resulting standardised scores (M = 100, [4]) were calculated. Phonological memory skills were assessed via a pseudoword repetition task [5]. Participants listened to 28 Spanish pseudowords, 4-18 phonemes long, in random order and repeated them one by one as fast and accurately as possible. After each repetition, they clicked on the button 'Send your response' to proceed to the next trial. Production of each pseudoword was checked offline for accuracy: 1 point score was assigned to each correctly pronounced target and a 0.25-point penalty per each incorrect or missed phoneme (min score: 0). The final phonological memory score was computed as total accuracy considering the penalties.

References

[1] G. Piazza, C. D. Martin, and M. Kalashnikova, "The Acoustic Features and Didactic Function of Foreigner-Directed Speech: A Scoping Review," *Journal of Speech, Language, and Hearing Research*, Aug. 2022, doi: 10.1044/2022_JSLHR-21-00609.

[2] M. Uther, M. A. Knoll, and D. Burnham, "Do you speak E-NG-L-I-SH? A comparison of foreigner- and infant-directed speech," *Speech Communication*, vol. 49, no. 1, Art. no. 1, Jan. 2007, doi: 10.1016/j.specom.2006.10.003.

[3] G. Piazza, M. Kalashnikova, L. Fernández-Merino, and C. Martin, "Speakers' communicative intentions lead to acoustic adjustments in native and non-native directed speech." PsyArXiv, May 15, 2023. doi: 10.31234/osf.io/kz72c.

[4] A. S. Kaufman and N. L. Kaufman, "Kaufman Brief Intelligence Test, Second Edition," in *Encyclopedia of Special Education*, John Wiley & Sons, Ltd, 2014. doi: 10.1002/9781118660584.ese1325.

[5] S. E. Gathercole, C. S. Willis, A. D. Baddeley, and H. Emslie, "The Children's Test of Nonword Repetition: a test of phonological working memory," *Memory*, vol. 2, no. 2, Art. no. 2, Jun. 1994, doi: 10.1080/09658219408258940.
